# 706 Mobilization with Femoral Catheters in the Burn ICU: Retrospective Review of Practice Guidelines

**DOI:** 10.1093/jbcr/irae036.251

**Published:** 2024-04-17

**Authors:** Audrey M O'Neil, Cassandra Rush, Allison N Boyd, David Roggy, Brett C Hartman

**Affiliations:** Richard M. Fairbanks Burn Center, Indianapolis, IN; Eskenazi Health, Indianapolis, IN; Eskenazi Health, Zionsville, IN; Richard M. Fairbanks Burn Center at Eskenazi Health, Indianapolis, IN; Indiana University, Indianapolis, IN; Richard M. Fairbanks Burn Center, Indianapolis, IN; Eskenazi Health, Indianapolis, IN; Eskenazi Health, Zionsville, IN; Richard M. Fairbanks Burn Center at Eskenazi Health, Indianapolis, IN; Indiana University, Indianapolis, IN; Richard M. Fairbanks Burn Center, Indianapolis, IN; Eskenazi Health, Indianapolis, IN; Eskenazi Health, Zionsville, IN; Richard M. Fairbanks Burn Center at Eskenazi Health, Indianapolis, IN; Indiana University, Indianapolis, IN; Richard M. Fairbanks Burn Center, Indianapolis, IN; Eskenazi Health, Indianapolis, IN; Eskenazi Health, Zionsville, IN; Richard M. Fairbanks Burn Center at Eskenazi Health, Indianapolis, IN; Indiana University, Indianapolis, IN; Richard M. Fairbanks Burn Center, Indianapolis, IN; Eskenazi Health, Indianapolis, IN; Eskenazi Health, Zionsville, IN; Richard M. Fairbanks Burn Center at Eskenazi Health, Indianapolis, IN; Indiana University, Indianapolis, IN

## Abstract

**Introduction:**

Femoral catheters are a known barrier for ICU therapy sessions and mobility progression due to the anatomical location and potential risk of complications. Mobilization with femoral catheters has been studied in ICU populations, but has not been explored specifically within the burn population. A previous case series was completed in 2022, examining the safety of mobilization with femoral catheters in the burn ICU, following a change in practice guidelines. The purpose of this study is to further examine outcomes and complication rates following implementation of femoral catheter mobilization guidelines, as well as retrospectively compare data to outcomes prior to initiation of the mobilization protocol.

**Methods:**

Retrospective review was completed on 17 patients prior to and following implementation of new femoral catheter mobility guidelines within a 15-bed adult burn unit, 34 patients total. Burn therapy notes were reviewed for burn admissions with at least one femoral catheter in place, including arterial, central, and dialysis catheters. Demographic data, admission statistics, line placement timelines, and active mobility achieved during therapy sessions were recorded for both the non-mobilization (NMG) and mobilization groups (MG).

**Results:**

The 34 patients reviewed had 99 total lines placed (30 NMG, 69 MG). Mobilization restrictions limited the NMG group resulting in less therapy sessions (n=281), active mobility sessions (n=5), and mobility activities (n=6). Change in mobility protocols for the MG group resulted significantly more therapy sessions (n=516) and active mobility sessions (n=83), including 146 total mobility activities including transitions to chair mode of bed, cardiac chair, tilt table, sitting edge of bed (EOB), standing, active chair transfers, and cycle ergometry. Additionally, 82% (n=14) of patients in the MG participated in mobility earlier, while intubated, while the NMG was unable to participate in out of bed activity while intubated. No catheter associated adverse events occurred during active mobility sessions and no complications were associated with participation in mobility.

**Conclusions:**

Establishing femoral catheter mobility guidelines allowed patients with femoral catheters to participate in significantly greater active therapy sessions, compared to previously restricted groups, without compromising safety. Early mobilization within ICU settings is necessary in preventing complications, combating muscle wasting, and improving long term outcomes for survivors. This study further supports that the presence of femoral catheters alone should not limit the progression of mobility interventions.

**Applicability of Research to Practice:**

Therapy driving mobilization guidelines are safe within the burn ICU setting, when proper training and staff resources are present. Change in practice guidelines allowed significant improvements in patient access to active therapy interventions.

